# *cjrABC-senB* hinders survival of extraintestinal pathogenic *E. coli* in the bloodstream through triggering complement-mediated killing

**DOI:** 10.1186/s12929-020-00677-4

**Published:** 2020-08-06

**Authors:** Wen-Chun Huang, Yi-Jyun Liao, Masayuki Hashimoto, Kuan-Fu Chen, Chishih Chu, Po-Chuen Hsu, Shuying Wang, Ching-Hao Teng

**Affiliations:** 1 Institute of Molecular Medicine, College of Medicine, National Cheng Kung University, 4th F, 367 Sheng Li Road, North District, Tainan City, Taiwan; 2 Institute of Basic Medical Sciences, College of Medicine, National Cheng Kung University, Tainan City, Taiwan; 3 Center of Infectious Disease and Signaling Research, National Cheng Kung University, Tainan City, Taiwan; 4 Department of Microbiology, Immunology, and Biopharmaceuticals, National Chiayi University, Chiayi City, Taiwan; 5 Department of Microbiology and Immunology, College of Medicine, National Cheng Kung University, Tainan City, Taiwan; 6 Department of Biotechnology and Bioindustry Sciences, College of Bioscience and Biotechnology, National Cheng Kung University, Tainan City, Taiwan

**Keywords:** Extraintestinal pathogenic *E. coli*, *cjrA*, *cjrB*, *cjrC*, *senB*, *cjrABC-senB*, ExPEC, Urinary tract infections, Bacteremia

## Abstract

**Background:**

Extraintestinal pathogenic *E. coli* (ExPEC) is a common gram-negative organism causing various infections, including urinary tract infections (UTIs), bacteremia, and neonatal meningitis. The *cjrABC-senB* gene cluster of *E. coli* contributes to ExPEC virulence in the mouse model of UTIs. Consistently, the distribution of *cjrABC-senB* is epidemiologically associated with human UTIs caused by *E. coli*. *cjrABC-senB*, which has previously been proposed to encode an iron uptake system, may facilitate ExPEC survival in the iron availability-restricted urinary tract. Given that the bloodstream is also an iron limited environment to invading bacteria, the pathogenic role of *cjrABC-senB* in ExPEC bacteremia, however, remains to be investigated.

**Methods:**

The ability of ExPEC RS218 strains with and without *cjrABC-senB* to survive in the mouse bloodstream and human serum was evaluated. Subsequently, the role of this gene cluster in the ExPEC interaction with the complement system was evaluated. Finally, the distribution of *cjrABC-senB* in human clinical *E. coli* isolates was determined by PCR. The frequency of *cjrABC-senB* in bacteremia isolates that were not associated with UTIs (non-UTI bacteremia isolates) was compared with that in UTI-associated isolates and fecal isolates.

**Results:**

Expression of *cjrABC-senB* attenuated the survival of RS218 in the mouse bloodstream and human serum. The *cjrABC-senB*-harboring strains triggered enhanced classical- and alternative-complement pathway activation and became more vulnerable to complement-mediated killing in serum. *cjrA* was identified as the major gene responsible for the attenuated serum survival. Expressing *cjrABC-senB* and *cjrA* increased bacterial susceptibility to detergent and induced periplasmic protein leakage, suggesting that the expression of these genes compromises the integrity of the outer membrane of ExPEC. In addition, the frequency of *cjrABC-senB* in non-UTI bacteremia isolates was significantly lower than that in UTI-associated isolates, while the frequencies in non-UTI bacteremia isolates and fecal isolates showed no significant difference. Consistently, this epidemiological investigation suggests that *cjrABC-senB* does not contribute to *E. coli* bacteremia in humans.

**Conclusion:**

The contribution of *cjrABC-senB* to the pathogenesis of ExPEC is niche dependent and contradictory because the genes facilitate ExPEC UTIs but hinder bacteremia. The contradictory niche-dependent characteristic may benefit the development of novel strategies against *E. coli*-caused infections.

## Background

Extraintestinal pathogenic *E. coli* (ExPEC) is one of the major causes of extraintestinal infections, such as urinary tract infections (UTIs), bacteremia, and neonatal meningitis [1]. Antibiotic treatment is the traditional measure used to treat *E. coli*-caused infections. However, the rapid emergence of antibiotic-resistant strains has become a critical issue for managing these infections [1–3]. The development of novel antimicrobial strategies is desperately needed. Given that bacterial virulence factors are potential targets for developing such strategies, understanding the roles of the virulence factors and the ways they facilitate infections are fundamental.

The *E. coli cjrABC-senB* gene cluster, which contains the genes *cjrA*, *cjrB*, *cjrC*, and *senB*, is located on virulence plasmids of many ExPEC strains and has been shown to contribute to the uropathogenesis of ExPEC [4–6]. It is proposed that the gene cluster facilitates iron uptake by ExPEC in the urinary tract, where the availability of iron is low [4, 7, 8], because the *cjrABC* genes are predicted to encode iron uptake factors. CjrA is homologous to a *Pseudomonas aeruginosa* protein, PhuW, which is important for heme uptake. CjrB is homologous to TonB proteins of various bacteria, while CjrC is homologous to TonB-dependent outer membrane heme/hemoglobin or siderophore receptors [5, 9]. It has been noted that many ExPEC virulence factors contribute to infections in different tissues, while some contribute to infection in only specific tissues [1]. As bacterial survival in the bloodstream is a critical step for ExPEC to cause lethal systemic infections, it is of interest to elucidate the role of *cjrABC-senB* in ExPEC survival in the bloodstream, where iron availability is restricted for invading pathogens [10, 11].

The complement system is the first line of innate defense in the bloodstream against invading pathogens. The complement system can be activated by three distinct pathways: the classical, alternative, and lectin pathways [12]. Activation of the three pathways leads to the production and deposition of the complement protein C3b on the surface of invading pathogens. C3b deposition triggers the activation of the downstream common terminal complement pathway, resulting in the formation of membrane attack complexes (MACs) on the pathogen surface, thus killing the pathogen. The direct binding or indirect, antibody-dependent binding of C1q to invading pathogens can trigger the classical pathway (CP). The alternative pathway (AP) starts with the spontaneous hydrolysis of C3 to produce C3b, while the lectin pathway (LP) is initiated by the binding of mannose-binding lectin to the carbohydrate structures on the pathogen surface.

In this study, we found that ExPEC expressing the *cjrABC-senB* operon induced a significantly lower level of bacteremia in a mouse bacteremia model than cognate strains without this operon. Our results suggest that the complement system in the bloodstream is responsible for the decreased survival of *cjrABC-senB*-harboring pathogens. The detailed mechanism was further elucidated within this study.

## Methods

### Bacterial strains and plasmids

*E. coli* K1 strain RS218 (O18:K1:H7) is a bacteremia clinical isolates which is isolated from the cerebrospinal fluid (CSF) from a neonate with meningitis [13–16]. The spontaneous streptomycin-resistant derivative of RS218 and its derivatives were used in this study (Table 1). The RS218 mutants were constructed by a PCR-based method described previously [20, 21] (Table 1). The low-copy-number plasmid pCL1920 was utilized to clone *cjrABC-senB* and the individual genes in this gene cluster (Table 1).

**Table 1:** *E. coli* strains and plasmids used in this study

Strains or plasmids	Relevant information	Sources
***E. coli*Strains**		
RS218 (WT-RS218)	*E. coli* K1 RS218 isolated from the cerebrospinal fluid of a neonate with meningitis.	[16, 17]
*∆lacZ*-RS218	RS218 with a *lacZ* deletion (The otherwise WT-RS218)	[18]
*∆cjr*-RS218	RS218 with a *cjrABC-senB* deletion	This study
*∆cjr∆lacZ*-RS218	*∆cjr*-RS218 with a *lacZ* deletion	This study
Cjr^+^-RS218	*∆cjr*-RS218 harboring pCL1920-*cjrABC-senB*	This study
Cjr^+^-*∆lacZ*-RS218	Cjr^+^-RS218 with a *lacZ* deletion	This study
Cjr^−^-RS218	*∆cjr*-RS218 harboring pCL1920	This study
CjrA-RS218	*∆cjr*-RS218 harboring pCL1920-*cjrA*	This study
CjrB-RS218	*∆cjr*-RS218 harboring pCL1920-*cjrB*	This study
CjrC-RS218	*∆cjr*-RS218 harboring pCL1920-*cjrC*	This study
SenB-RS218	*∆cjr*-RS218 harboring pCL1920-*senB*	This study
**Plasmids**		
pCL1920	Low-copy-number plasmid	[19]
pCL1920-*cjrABC-senB*	pCL1920 harboring *cjrABC-senB*, which is under the control of the *lac* promoter on the plasmid	This study
pCL1920-*cjrA*	pCL1920 harboring *cjrA* with a His_6_-tagged 3′-end, which is under the control of the *lac* promoter on the plasmid.	This study
pCL1920-*cjrB*	pCL1920 harboring *cjrB* with a His_6_-tagged 3′-end, which is under the control of the *lac* promoter on the plasmid.	This study
pCL1920-*cjrC*	pCL1920 harboring *cjrC* with a His_6_-tagged 3′-end, which is under the control of the *lac* promoter on the plasmid.	This study
pCL1920-*senB*	pCL1920 harboring *senB* with a His_6_-tagged 3′-end, which is under the control of the *lac* promoter on the plasmid.	This study

The bacteremia *E. coli* isolates that were not associated with UTIs and biliary tract infections (BTIs) were collected in National Cheng Kung University Hospital between October and December of 2005.

### Human sera, and C1q

The normal human serum (NHS) used in this study was pooled from the serum of 8 healthy adults and stored in aliquots at − 80 °C. Heat-inactivated NHS (HI-NHS) was prepared by heating the NHS at 56 °C for 30 min. The C1q-depleted and factor B-depleted sera (Calbiochem) supplemented with 5 mM CaCl_2_ and 2 mM MgCl_2_ and then diluted with PBS were served and CP- and AP- blocked sera. In addition, heat-inactivated C1q anti-serum or heat-inactivated properdin anti-serum (Calbiochem, Darmstadt, Germany, and Sigma-Aldrich, St. Louis, MO) were added to the final concentrations of 10 and 15% in 40% NHS diluted with PBS to block CP and AP respectively [22, 23] . To inhibit the LP pathway, NHS was treated with 100 mM mannose [24]. The purified C1q protein was purchased from Complement Technology, Inc. (Tyler, TX).

### In vivo complement depletion with cobra venom factor (CVF)

Complement depletion in mice with cobra venom factor CVF) was performed as describe previously [25]. Briefly, CVF (Quidel) was diluted in PBS at a concentration of 2.5 U/100 μl. Mice (*n* = 15) were intraperitoneally injected with 5 U of CVF twice with an interval of 6 h. In the control group, animals (n = 15) were injected with 200 μl of PBS instead of CVF. At 48 h after the last CVF/PBS injection, the animals were subjected to co-infection experiments. Before the infection experiments, the levels of the CVF treatment-induced complement depletion was determined by the 50% haemolytic complement (CH_50_) activity of serum as previously described [26, 27]. Briefly, 200 μl mouse serum was serially diluted 2-fold with Veronal Buffered Saline (VBS) and then was incubated with 200 μl rabbit red blood cells (RBC) in VBS at 37 °C for 30 min. The intact RBC were pelleted by centrifugation at 1500 g for 5 min and O.D._540_ of the supernatant was measured. The percentage of hemolysis was used the following formula: hemolysis (%) = [(A - B)/(C - B)] × 100%. A is the O.D._540_ reading of the RBC incubated with CVF- or PBS-treated serum, B is the O.D._540_ reading of the RBC incubated with VBS buffer only, and C is the O.D._540_ nm reading of the RBC incubated with H_2_O. H_2_O induced the lysis of all the RBC. The lysis percentage induced by the CVF-treated and PBS-treated sera were plotted against dilution factors. Based on the resulting graphs, the dilutions required for 50% hemolysis (CH_50_) of CVF- and PBS- treated serum were calculated. The CH_50_ of the CVF-treated serum was approximately 15% of CH50 of the PBS-treated serum, indicating that CVF treatment induced 85% of complement depletion in the animals, compared to the PBS treated ones.

### The mouse model of *E. coli* bacteremia

To determine the role of *cjrABC-senB* in ExPEC bacteremia, equal numbers (1 × 10^7^ CFU) of the *E. coli* strains with or without these genes were co-inoculated or independently inoculated into 8-week-old BALB/c mice (*n* = 15) thorough intraperitoneal injection as previously described [18]. At 14 h post-infection, the bacterial blood counts were determined. In the co-inoculation experiments (*n* = 10), the two bacterial strains were differentiated by the colors of their colonies (blue and white) after cultivation on LB agar containing 0.5 mM IPTG and 20 μg/ml X-gal.

### Preparation of IgG-depleted serum

To remove serum IgG, 1 ml of 40% NHS diluted with phosphate-buffered saline (PBS) was incubated with 0.6 ml of recombinant protein G-Sepharose (Invitrogen, Grand Island, NY) at 4 °C for 1 h. The IgG depleted serum was obtained from the supernatant by centrifugation at 1500 g for 10 min at 4 °C. More than 90% of the IgG was removed in the depleted serum based on Western blot analysis (data not shown).

### Serum survival assay

For the serum survival assays with WT-RS218 and ∆*cjr*-RS218 (Table 1), 20 μl of the overnight bacterial culture was inoculated in 2 ml of fresh LB medium containing 200 μM 2, 2′-dipyridyl (DIP), a high affinity of iron chelator, at a ratio of 1:100 for 2 h to induce the expression of *cjrABC-senB* [28]. For the assays performed with the *E. coli* strains transformed with the empty vector pCL1920 or the plasmids harboring the *cjrABC-senB* genes (Table 1), overnight cultures of the bacteria were used. These strains didn’t need DIP treatment because the *cjrABC-senB* genes in the plasmids were under the control of the *lac* promoter provided by the plasmid. The leakage of the *lac* promoter allowed the constitutive expression of these genes. For the serum survival experiment, *E. coli* (1 × 10^6^ CFU) cells were incubated at 37 °C in 100 μl of 40% serum (NHS, HI-NHS, or modified serum) diluted with PBS. After different time periods of incubation, the live bacteria counts were determined by plating the solution on LB agar.

### Flow cytometry analysis

Bacteria (3 × 10^6^ CFU/ml) were incubated at 37 °C in 40% human sera (NHS, HI-NHS, or modified sera with and without heat inactivation) diluted with veronal buffer (Lonza, Walkersville, MD) or in 100 μl of veronal buffer containing 25 μg/ml of C1q protein for different time periods. The levels of the serum components deposited on bacteria were determined by probing with the corresponding primary and secondary antibodies as previously described [18, 23] and then analyzed on a FACSCalibur™ flow cytometer (Becton-Dickinson).

### Purification of bacterial outer and inner membrane fractions and lipopolysaccharides

The bacterial inner and outer membrane fractions were separated using the detergent sodium lauryl sarcosinate as previously described [29]. The lipopolysaccharides (LPS) on the *E. coli* strains were purified according to methods described by Kariyawasam et al. [30].

### Ribonuclease (RNase) leakage assay

The overnight bacterial culture was adjusted to OD_600_ = 0.2 in PBS. Then, 10 μl of the culture was added to LB agar plates containing 2.5% (w/w) Toluidine blue O and 0.2% (w/w) yeast RNA. After incubation for 2 days at 37 °C, pink halos around the bacterial colony on the agar were observed because of the RNase leakage from the periplasm of bacteria [31–33].

### Distribution rate of *cjrABC-senB* in the bacteremia *E. coli* isolates that were not associated with UTIs and BTIs

The frequency of *cjrABC-senB* in the in the bacteremia *E. coli* isolates that were not associated with UTIs and BTIs was determined by PCR as described previously [5].

### Statistical analysis

For the mouse model of *E. coli* bacteremia, the coinfection results were analyzed using a nonparametric Wilcoxon matched-pair test, while the independent infection results were analyzed used non-parametric Mann-Whitney test [34]. Comparisons involving the distribution rates of *cjrABC-senB* in different groups were measured by using two-tailed Fisher’s exact test. For the rest of the experiments, Student’s t-test was used. A *P* value of < 0.05 was set as the threshold for statistical significance.

## Results

### Deletion of *cjrABC-senB* increases ExPEC’s ability to cause bacteremia

**Fig. 1:**
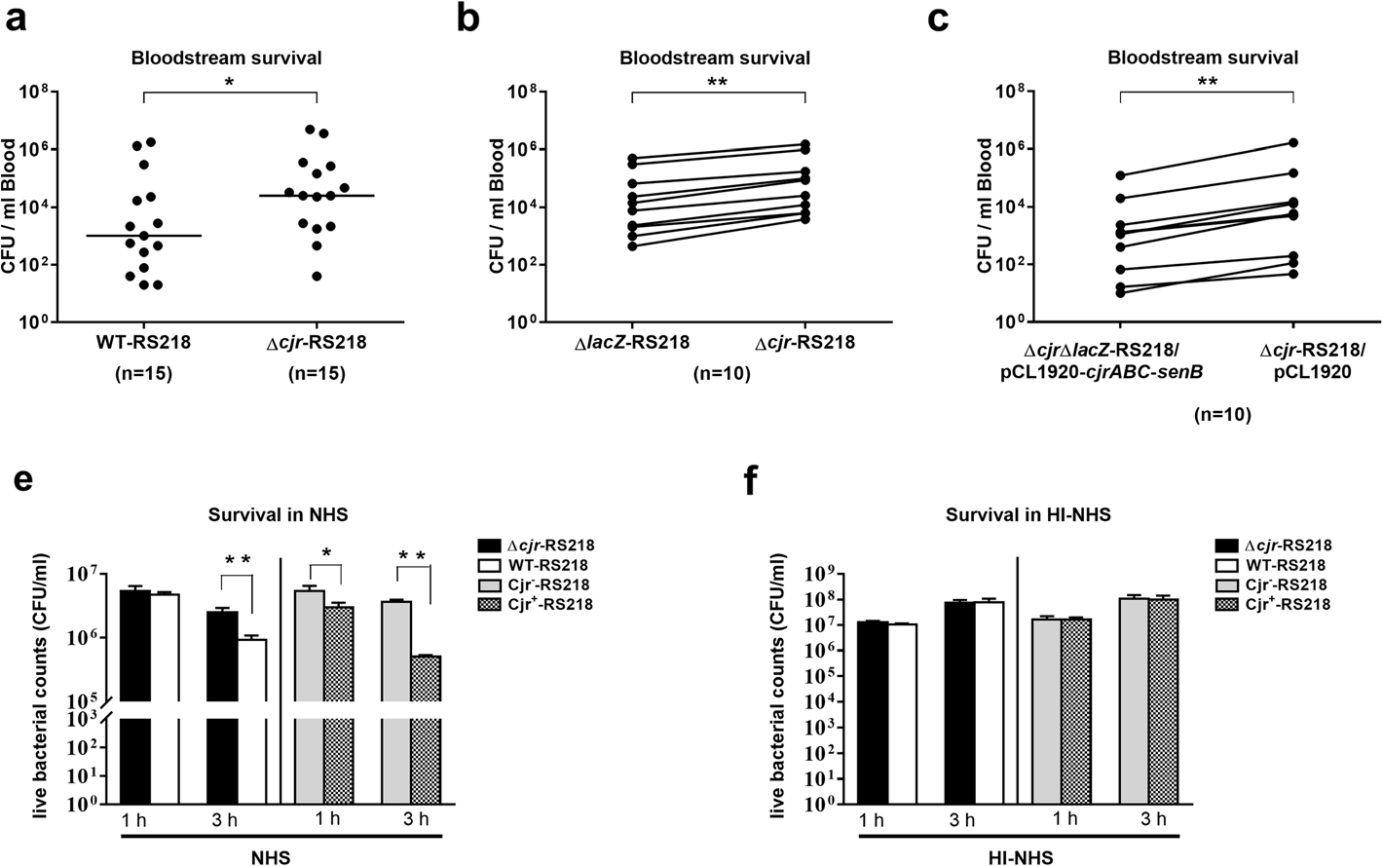
The survival of RS218 strains with and without *cjrABC-senB* in the mouse bloodstream and human serum. (**a**) Independent infections of mice with WT-RS218 and *∆cjr*-RS218 (1 × 10^7^ CFU/mouse). Bacterial blood counts were determined at 14 h post-inoculation. (**b**) Coinfection of mice with equal numbers of *∆cjr*-RS218 and *∆lacZ*-RS218 (1 × 10^7^ CFU/strain/mouse). Bacterial blood counts were determined at 14 h post-inoculation. (**c**) Coinfection of mice with equal numbers of *∆cjr*-RS218/pCL1920 and ∆*cjr*∆*lacZ*-RS218/pCL1920-*cjrABC-senB*. Blood counts were determined at 14 h post-infection. The horizontal bars represent the median values. For (**b**) and (**c**) the bacterial counts derived from same animals are connected with lines. (**d**) and (**e**) The serum survival of the RS218 strains with and without *cjrABC-senB* (1 × 10^7^ CFU/ml) after 1 h and 3 h incubation in 40% NHS (**d**) and 40% HI-NHS(**e**). The results are shown as the means ± standard deviations, and the data are representative of three independent experiments performed in triplicate. *, *P* value < 0.05; **, *P* value < 0.01; ***, *P* value < 0.001

To investigate whether *cjrABC-senB* plays a role in ExPEC bacteremia, we utilized the archetypal K1 bacteremia *E. coli* strain RS218 [14], which harbors a copy of *cjrABC-senB* encoded in the plasmid pRS218 [6]. Equal numbers of the wild-type RS218 (WT-RS218) and a *cjrABC-senB* deletion mutant of RS218 (∆*cjr*-RS218) were independently inoculated into mice through intraperitoneal injection. At 14 h post-inoculation the blood counts of the bacteria were determined. As shown in Fig. 1a, the bacterial blood counts of ∆*cjr*-RS218 were significantly higher than those of WT-RS218, suggesting that expression of *cjrABC-senB* decreases RS218 survival in the bloodstream.

In addition, we further investigated whether RS218 strains with and with *cjrABC-senB* also show different bloodstream survival when co-inoculated in mice. Equal amounts of ∆*cjr*-RS218 and the otherwise wild-type RS218 (∆*lacZ*-RS218) were intraperitoneally co-inoculated into mice, and the counts of the two strains in the bloodstream was determined at 14 h post-inoculation (*lacZ* deletion did not affect bloodstream survival of *E. coli*; data not shown). To differentiate and enumerate the bacteria in the bloodstream, blood samples from the infected animals were spread on LB agar containing IPTG and X-gal. The two strains can be differentiated by their colony colors because the colonies of *E. coli* harboring an intact *lacZ* (∆*cjr*-RS218) would be blue and those of the bacteria without *lacZ* (∆*lacZ*-RS218) would be white on the agar. Consistent with the results of the independent inoculation, *∆cjr*-RS218 outcompeted ∆*lacZ*-RS218 in the blood (Fig. 1b). Then, we performed a complementary experiment. Equal numbers of the ∆*cjr*-RS218 strain harboring the low-copy-number plasmid vector pCL1920 and the ∆*cjr*-RS218 strain with a *lacZ* deletion (∆*cjr*∆*lacZ*-RS218; Table 1) harboring the plasmid encoding *cjrABC-senB* (pCL1920-*cjrABC-senB*; Table 1) were inoculated intraperitoneally into animals, and the blood counts of each bacteria were determined at 14 h post-inoculation by plating on LB agar containing IPTG and X-gal. Consistently, the strain without *cjrABC-senB* outcompeted the strain with this gene cluster (Fig. 1c). These results further confirmed that the expression of *cjrABC-senB* hinders bacterial survival in the bloodstream.

Because serum-mediated killing is one of the major defenses against invading bacterial pathogens in the bloodstream, we investigated whether *cjrABC-senB* is involved in the serum survival of RS218. RS218 strains with and without *cjrABC-senB* were independently cultured in 40% normal human serum (NHS) or 40% heat-inactivated NHS (HI-NHS). After 1 h and 3 h incubation, the counts of live bacteria were determined. WT-RS218 showed significantly lower survival than ∆*cjr*-RS218 after 3 h of incubation in NHS (Fig. 1d). A complementary experiment showed consistent results. The ∆*cjr*-RS218 strain trans-complemented with pCL1920-*cjrABC-senB* (this strain was designated Cjr^+^-RS218) showed significantly lower survival than the ∆*cjr*-RS218 strain harboring the plasmid vector pCL1920 (this strain was designated Cjr^−^-RS218) after 1 h and 3 h incubation in NHS (Fig. 1d). These results suggest that expressing *CjrABC-senB* attenuates ExPEC survival in NHS.

However, in HI-NHS, the *cjrABC-senB*-harboring strains showed survival levels similar to those of the corresponding strains without this gene cluster (Fig. 1e). Given that the complement system plays an important role in resisting invading bacteria in the serum and that the function of this system is heat labile, the results suggest that the complement system may be responsible for the differential killing of the ExPEC strains with and without the *CjrABC-senB* gene cluster.

### *cjrABC-senB*-expressing ExPEC encounters a stronger complement attack in NHS than *cjrABC-senB*-deficient ExPEC

**Fig. 2:**
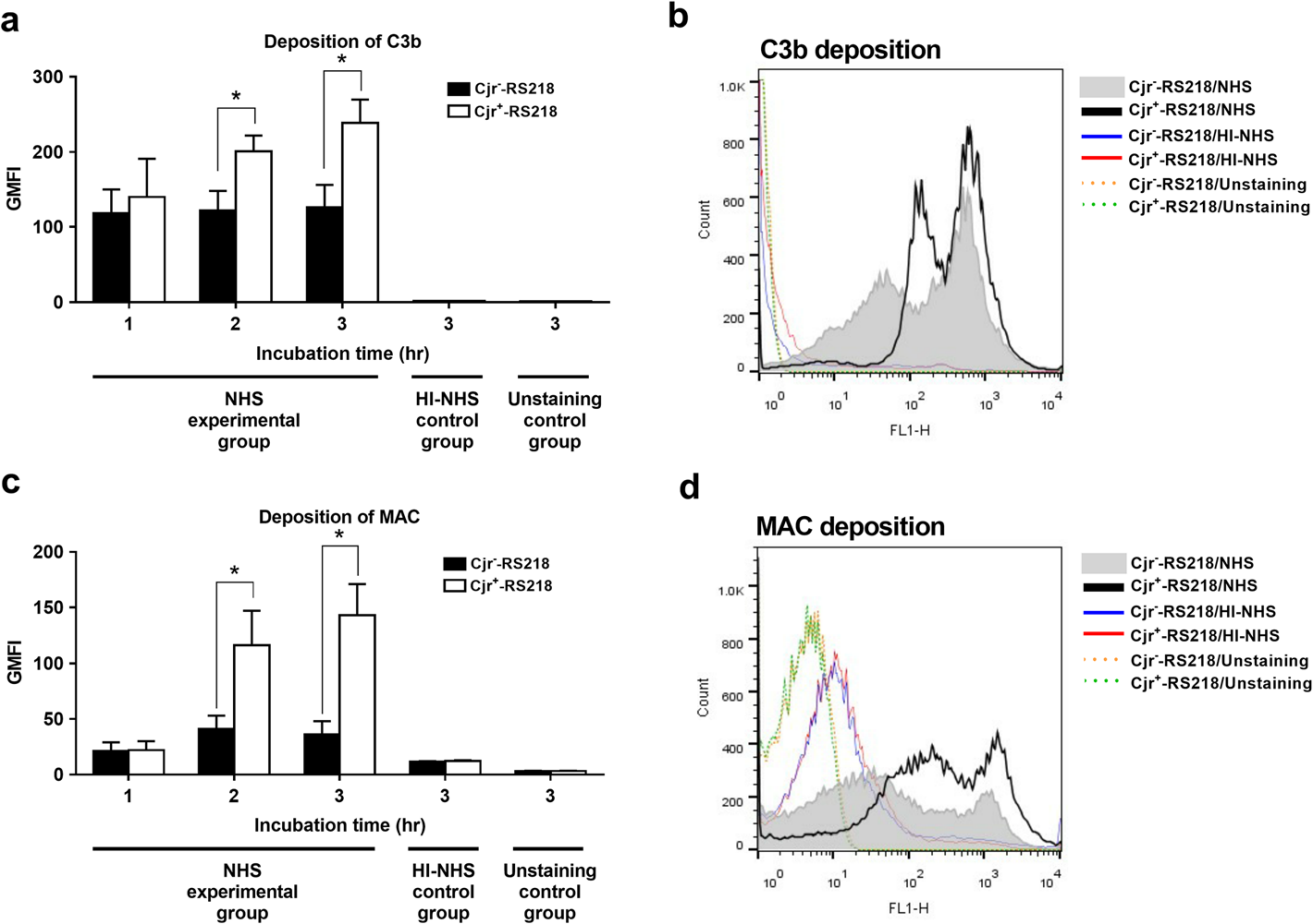
Deposition of C3b and MAC on Cjr^+^-RS218 and Cjr^−^-RS218 in 40% NHS for various periods. (**a**) Levels of C3b deposition on Cjr^+^-RS218 and Cjr^−^-RS218 after incubation with NHS for the indicated time periods. (**b**) Flow cytometry histogram of C3b deposition on the bacteria after 3 h of incubation in NHS. (**c**) Levels of MAC deposition on Cjr^+^-RS218 and Cjr^−^-RS218 after incubation with NHS for the indicated time periods. (**d**) Flow cytometry histogram of MAC deposition on the bacteria after 3 h of incubation in NHS. For (**a**) and (**c**) The data are presented with geometric mean fluorescence intensity (GMFI). The HI-NHS control groups were bacteria incubated in HI-NHS, while the unstaining control groups were the bacteria without fluorescence staining. The results are shown as the means ± standard deviations, and the data are representative of three independent experiments performed in triplicate. *, *P* value < 0.05

In serum, the levels of C3b and MAC deposition on bacteria reflect the intensity of complement activation that occurs on the bacterial surface [18]. To investigate whether ExPEC with and without *cjrABC-senB* encounter different levels of complement-mediated attack in the serum, the levels of C3b and MAC deposition on Cjr^+^-RS218 and Cjr^−^-RS218 were measured by flow cytometry after incubation with NHS. As shown in Fig. 2a-2d, after incubation in 40% NHS for 2 and 3 h, the levels of C3b and MAC deposition on Cjr^+^-RS218 were significantly higher than those on Cjr^−^-RS218, indicating that the presence of *cjrABC-senB* triggers a stronger complement-mediated attack on the bacteria, which may be responsible for the attenuated ExPEC serum survival due to the expression of this gene cluster.

### The CP and AP are responsible for the decreased serum survival of the *cjrABC-senB-*expressing ExPEC

**Fig. 3:**
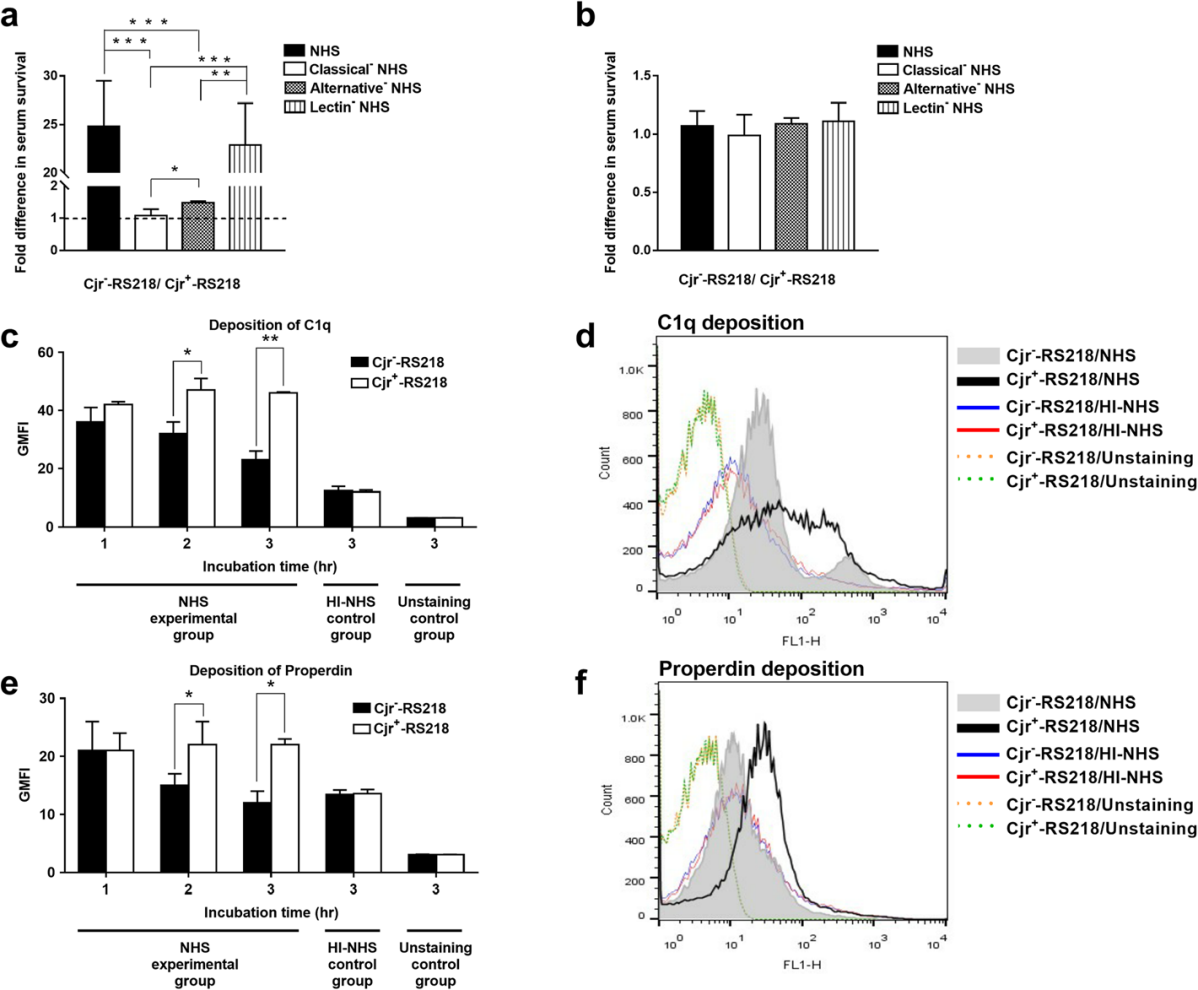
The fold difference in the serum survival of Cjr^−^-RS218 compared to that of Cjr^+^-RS218 and the deposition of C1q and properdin on the strains. (**a**) The fold difference in the serum survival (the survival rate of Cjr^−^-RS218/the survival rate of Cjr^+^-RS218) after 3 h of incubation in 40% sera, which were NHS and NHS with the classical, alternative, or lectin pathway inhibited. The horizontal dashed line represents 1-fold (the survival of the two strains is similar). The serum survival of Cjr^−^-RS218 was approximately 24.8-, 1.1-, 1.5-, and 22.9-fold greater than that of Cjr^+^-RS218 in NHS or NHS in which the classical, alternative, or lectin pathway was inhibited, respectively. (**b**) The fold difference in survival in 40% HI-NHS and 40%-modified HI-NHS, in which the classical, alternative, or lectin pathway was inhibited. Cjr^−^-RS218 and Cjr^+^-RS218 showed similar serum survival in these sera. For (**a**) and (**b**), the results are shown as the mean ± standard deviation, and the data are representative of three independent experiments performed in triplicate. Classical^−^ NHS, C1q-depleted NHS in which the CP is blocked; Alternative^−^ NHS, factor B-depleted NHS in which the AP is blocked; Lectin^−^ NHS, mannose-treated NHS in which the LP is blocked; Classical^−^ HI-NHS, heat-inactivated C1q-depleted NHS; Alternative^−^ HI-NHS, heat-inactivated factor B-depleted NHS; Lectin^−^ HI-NHS, heat-inactivated mannose-treated NHS. (**c**) The levels of C1q deposition after incubation in 40% NHS. (**d**) Flow cytometry histogram of C1q deposition on the bacteria after 3 h of incubation in NHS. (**e**) The levels of properdin deposition after incubation in 40% NHS. (**f**) Flow cytometry histogram of properdin deposition on the bacteria after 3 h of incubation in NHS. For (**c**) and (**e**), the data are presented with GMFI. The results are shown as the means ± standard deviations, and the data are derived from three independent experiments. The HI-NHS control groups were bacteria incubated in HI-NHS, while the unstaining control groups were the bacteria without fluorescence staining. *, *P* value < 0.05; **, *P* value < 0.01; ***, *P* value < 0.001

The roles of the three complement pathways in the differential killing of Cjr^+^-RS218 and Cjr^−^-RS218 were investigated. The strains were independently incubated in 40% NHS and 40% NHS with a blocked CP (C1q-depleted serum), AP (factor B-depleted serum), or LP (mannose treated serum). After 3 h of incubation, the survival rates of each strain in these sera were determined. Then, the survival rate of Cjr^−^-RS218 in a serum was compared with that of Cjr^+^-RS218 in the same kind of serum. As shown in Fig. 3a, the survival rates of Cjr^−^-RS218 were 24.8 ± 4.7, 1.1 ± 0.2, 1.5 ± 0.1, and 22.9 ± 4.3-fold greater than those of Cjr^+^-RS218 in normal (NHS), CP-blocked, AP-blocked, and LP-blocked sera, respectively. The fold difference of the bacterial survival in the CP- and AP-blocked sera was significantly lower than that in NHS, while the survival difference in NHS and the LP-blocked serum showed no statistical significance (Fig. 3a). When these sera were heat inactivated, Cjr^+^-RS218 and Cjr^−^-RS218 showed similar survival rates (Fig. 3b). In addition, it is known that anti-C1q and anti-properdin antibodies can block CP and AP, respectively [22, 23]. Consistently, the survival difference of Cjr^+^-RS218 and Cjr^−^-RS218 in the serum treated with anti-C1q antibody or anti-properdin antibody-treated sera was significantly lower that the difference in NHS (Additional file 1: Fig. S1). These results suggest that the complement-mediated differential killing of Cjr^+^-RS218 and Cjr^−^-RS218 in NHS is due to mainly CP- and AP-mediated bactericidal activity.

The above findings directed us to speculate that Cjr^+^-RS218 may trigger stronger CP- and AP-mediated complement activation than Cjr^−^-RS218. In NHS, C1q binding to the bacteria initiates CP activation, while properdin is a positive regulator and an initiator of the AP [35–37]. Thus, after incubation in serum, higher levels of C1q and properdin deposition on the bacterial surface indicate that higher levels of CP and AP activation are triggered by the bacteria [18, 37]. To investigate whether Cjr^+^-RS218 triggers higher levels of CP and AP activation than Cjr^−^-RS218 in NHS, the levels of C1q and properdin deposition on the bacteria were determined by flow cytometry after incubation with NHS. As shown in Fig. 3c, d, e, and f, Cjr^+^-RS218 exhibited significantly higher levels of C1q and properdin deposition than Cjr^−^-RS218 after 2 h and 3 h of incubation in 40% NHS, suggesting that expression of *cjrABC-senB* triggers stronger activation of the CP and AP in NHS.

In addition, it is known that ExPEC can actively suppress the activation of the complement system through recruiting host complement regulators on its surface. For example, ExPEC can recruit the CP regulator C4bp to block the activation of the CP [38], while the bacteria can recruit the AP regulator factor H (FH) to block the activation of the AP [39]. Thus, we investigated C4bp and FH deposition on Cjr^+^-RS218 and Cjr^−^-RS218. However, the strains showed similar deposition of the regulators (data not shown), suggesting that expression of *cjrABC-senB* does not affect ExPEC’s ability to recruit the complement regulators.

### Expression of *cjrABC-senB* triggers robust antibody-dependent CP activation

**Fig. 4:**
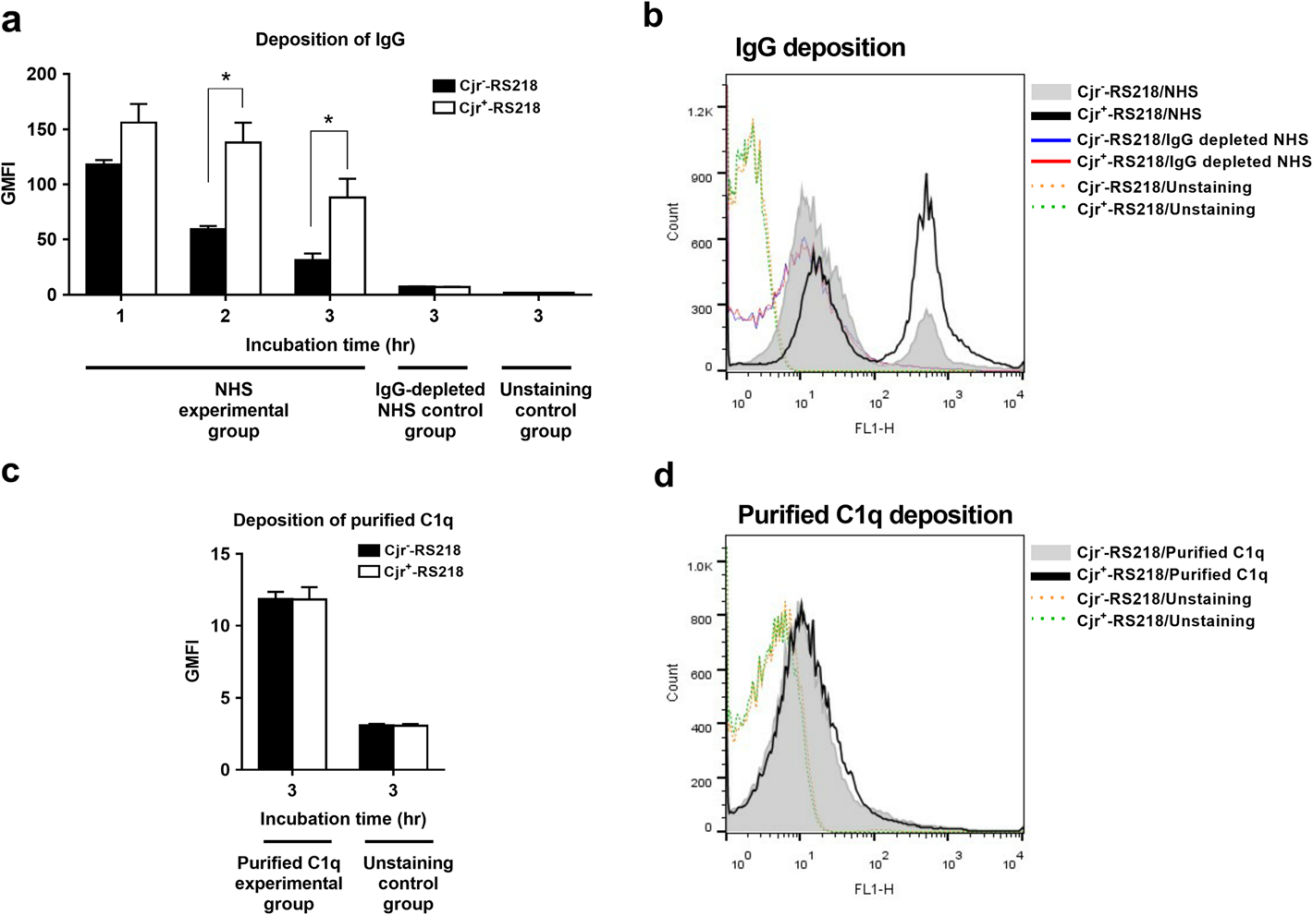
The deposition of serum IgG and purified C1q proteins on Cjr^+^-RS218 and Cjr^−^-RS218. (**a**) Levels of IgG deposition on the indicated strains after incubation in 40% NHS. (**b**) Flow cytometry histogram of IgG deposition on the bacteria after 3 h of incubation in 40% NHS. (**c**) Levels of purified C1q protein on the indicated strains after incubation with a solution containing 32 μg/ml C1q protein for 3 h. (**d**) Flow cytometry histogram of C1q deposition on the bacteria after incubation with a solution containing 32 μg/ml C1q protein for 3 h. For (**a**) and (**c**), the data are derived from flow cytometry analyses and presented as GMFI. The IgG depleted-NHS control group was bacteria incubated in IgG depleted-NHS, while the unstaining control groups were the bacteria without fluorescence staining. *, *P* value < 0.05

The CP can be activated through direct binding of C1q onto the bacteria or through antibody-dependent binding of C1q by binding of this component to the antibody that has already bound to the bacteria. We investigated whether the expression of *cjrABC-senB* affects antibody-independent and antibody-dependent C1q deposition by flow cytometry analyses. In NHS, Cjr^+^-RS218 exhibited a significantly higher level of IgG binding than Cjr^−^-RS218 (Fig. 4a and b). However, after incubation with purified C1q, the two strains showed similar levels of C1q deposition (Fig. 4c and d), in contrast to the above results showing that Cjr^+^-RS218 recruited higher levels of C1q deposition in NHS in which IgG is present (Fig. 3c and d). This result suggests that the expression of *cjrABC-senB* in ExPEC triggers stronger activation of the antibody-dependent CP.

### Expression of *cjrABC-senB* increases bacterial sensitivity to MAC-mediated attack

**Fig. 5:**
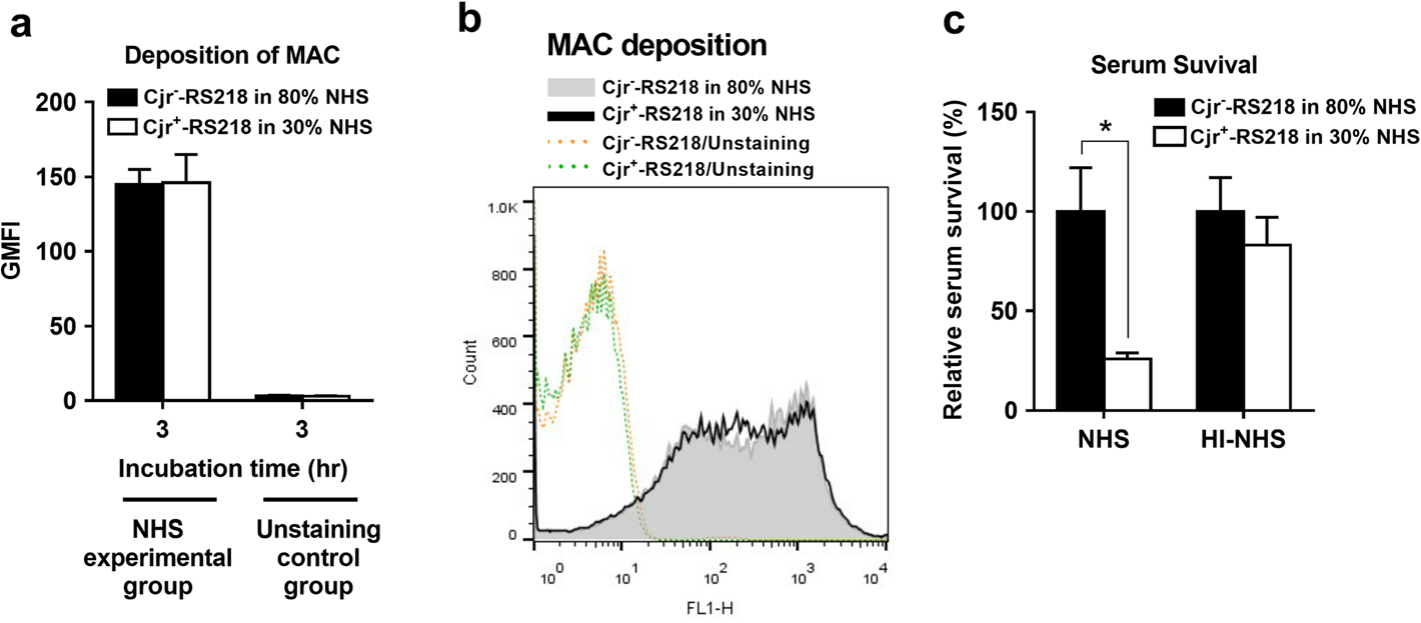
The survival of Cjr^−^-RS218 and Cjr^+^-RS218 under a similar level of MAC-mediated attack in human serum. (**a**) Cjr^−^-RS218 and Cjr^+^-RS218 showed a similar level of MAC binding to their surfaces after incubation with 80 and 30% NHS, respectively, for 3 h. The data are derived from flow cytometry analyses and presented as GMFI. The unstaining control group was the bacteria without fluorescence staining. (**b**) Flow cytometry histogram of MAC deposition on Cjr^−^-RS218 and Cjr^+^-RS218 after 3 h of incubation in 80 and 30% NHS, respectively. (**c**) The relative survival rates of Cjr^−^-RS218 and Cjr^+^-RS218 after incubation in 80% serum (NHS or HI-NHS) and 30% serum (NHS or HI-NHS), respectively, for 3 h. The data are shown as the relative survival rates compared with those of Cjr^−^-RS218. The results are shown as the means ± standard deviations, and the data are representative of three independent experiments performed in triplicate. *, *P* value < 0.05

To better understand why Cjr^+^-RS218 has lower serum survival, we further investigated whether Cjr^+^-RS218 is more vulnerable to the complement (MAC)-mediated attack than Cjr^−^-RS218 by assessing the survival of the two strains under the same level of MAC binding. For Cjr^+^-RS218 and Cjr^−^-RS218 to have a similar level of MAC deposition in the serum, we incubated the bacterial strains in different concentrations of NHS and determined the MAC deposition on the bacteria. We found that the MAC deposition level on Cjr^+^-RS218 incubated in 30% NHS was similar to that on Cjr^−^-RS218 incubated in 80% NHS (Fig. 5a and b), suggesting that Cjr^+^-RS218 in 30% NHS and Cjr^−^-RS218 in 80% NHS encounter a similar level of complement-mediated attack. As shown in Fig. 5c, the survival of Cjr^+^-RS218 in 30% NHS was significantly lower than that of Cjr^−^-RS218 in 80% NHS, suggesting that Cjr^+^-RS218 is more vulnerable (susceptible) to the complement-mediated attack than Cjr^−^-RS218.

### Bloodstream survival of RS218 strains with and without *cjrABC-senB* in cobra venom factor-treated mice

**Fig. 6:**
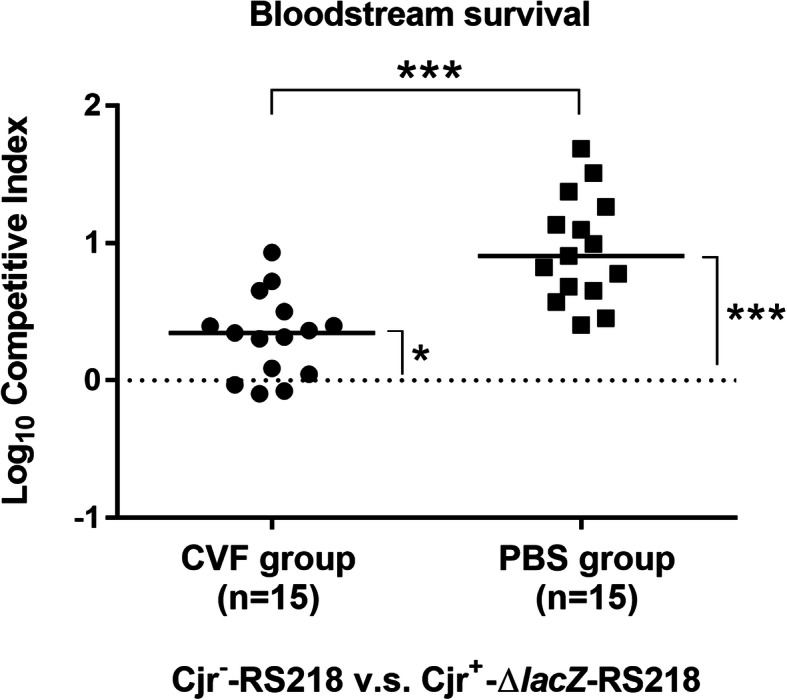
The survival of RS218 strains with and without *cjrABC-senB* in the bloodstream of CVF-treated mice and PBS-treated mice. Equal numbers of Cjr^−^-RS218 and Cjr^+^-∆*lacZ*-RS218 were co-inoculated into mice pretreated with CVF or PBS. At 14 h post-inoculation, the competitive index (CI) of Cjr^−^-RS218 v.s. Cjr^+^-*∆lacZ*-RS218 in the animals were determined. The CI was calculated as follows: CI = (bacterial counts of Cjr^−^-RS218/bacterial counts of Cjr^+^-*∆lacZ*-RS218)/ (inoculum counts of Cjr^−^-RS218/inoculum counts of Cjr^+^-*∆lacZ*-RS218). The horizontal bars represent the median values. *, *P* value < 0.05; ***, *P* value < 0.001

To investigate whether complement contributes to the differential killing of RS218 strains with and without cjrABC-senB in vivo, we used cobra venom factor (CVF) to deplete complement activity in mice. The CVF treatment depleted approximately 85% of the complement activity in sera (data not shown). Equal amounts of Cjr^−^-RS218 and Cjr^+^-*∆lacZ*-RS218 (the Cjr^+^-RS218 strain with a *lacZ* deletion) were co-inoculated into mice pretreated with CVF or PBS. At 14 h post-inoculation, the blood counts of the Cjr^−^ and Cjr^+^ bacteria were differentiated and determined by plating on LB agar containing IPTG and X-gal. The bacterial ratio (Cjr^−^ /Cjr^+^) in the blood was normalized by the ratio in inoculum to calculate the competitive (CI). The CI in the CVF-treated mice was significantly lower than that in the PBS-treated mice (Fig. 6). This result indicates that depletion of complement activity significantly decreases the survival difference between the Cjr^−^ and Cjr^+^ bacteria in the bloodstream, suggesting that complement contributes to the differential killing of RS218 strains with and without *cjrABC-senB* in vivo. Additionally, in the CVF-treated mice, the CI of Cjr^−^ v.s. Cjr^+^ was still higher than 1 in CVF-treated mice, suggesting that the Cjr^−^ bacteria still exhibits a higher survival than the Cjr^+^ bacteria in the animals. The residual complement activity or/and factors other than complement in the animals may contribute to the differential killing of the bacteria in the bloodstream.

### CjrA is responsible for the decreased serum survival of the *cjrABC-senB*-expressing ExPEC

**Fig. 7:**
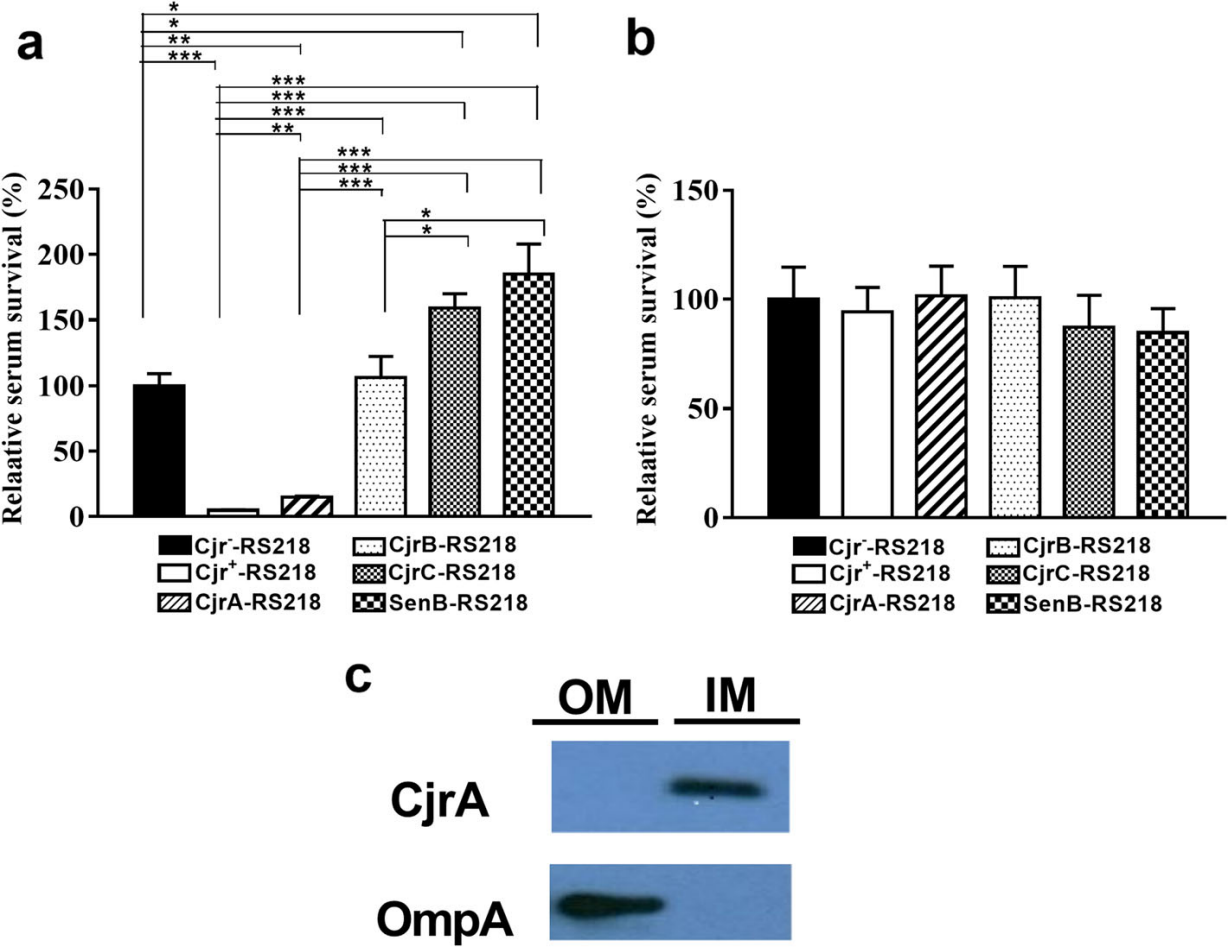
Survival of the RS218 strains harboring individual genes of the *cjrABC-senB* gene cluster in NHS or HI-NHS and localization of CjrA. (**a**) and (**b**) The survival rates of the bacterial strains were determined after the strains were incubated for 3 h in 40% NHS (**a**) or 40% HI-NHS (**b**). The data are shown as relative survival rates compared with those of Cjr^−^-RS218. The results are shown as the means ± standard deviations, which are representative of three independent experiments performed in triplicate. (**c**) The localization of CjrA. The outer and inner membrane (OM and IM) fractions of CjrA-RS218, which expresses C-terminally His_6_-tagged CjrA, were isolated and subjected to Western blot analysis with an anti-His_6_ antibody or OmpA antiserum. OmpA serves as an OM marker. *, *P* value < 0.05

Individual genes in *cjrABC-senB* were assessed for their contribution to the attenuated serum survival of ExPEC. The RS218 strains CjrA-RS218, CjrB-RS218, CjrC-RS218, and SenB-RS218, which express only CjrA, CjrB, CjrC, or SenB, respectively (Table 1), were analyzed for their serum survival. CjrA-RS218 showed a significantly lower survival rate in NHS than Cjr^−^-RS218, while the strains expressing the other genes showed similar or even slightly higher NHS survival rates than Cjr^−^-RS218 (Fig. 7a). On the other hand, they showed similar survival rates in HI-NHS (Fig. 7b). This finding suggests that CjrA is the major protein responsible for the decreased serum survival of *cjrABC-senB-*expressing ExPEC. Based on its sequence, CjrA is a potential inner membrane (IM) lipoprotein. We consistently found that the CjrA protein is located in the IM fraction of ExPEC (Fig. 7c), suggesting that expression of CjrA in the IM may indirectly interfere with outer membrane (OM) integrity, which affects the interaction between ExPEC and the complement system.

### The expression of *cjrABC-senB* compromises outer membrane integrity

**Fig. 8:**
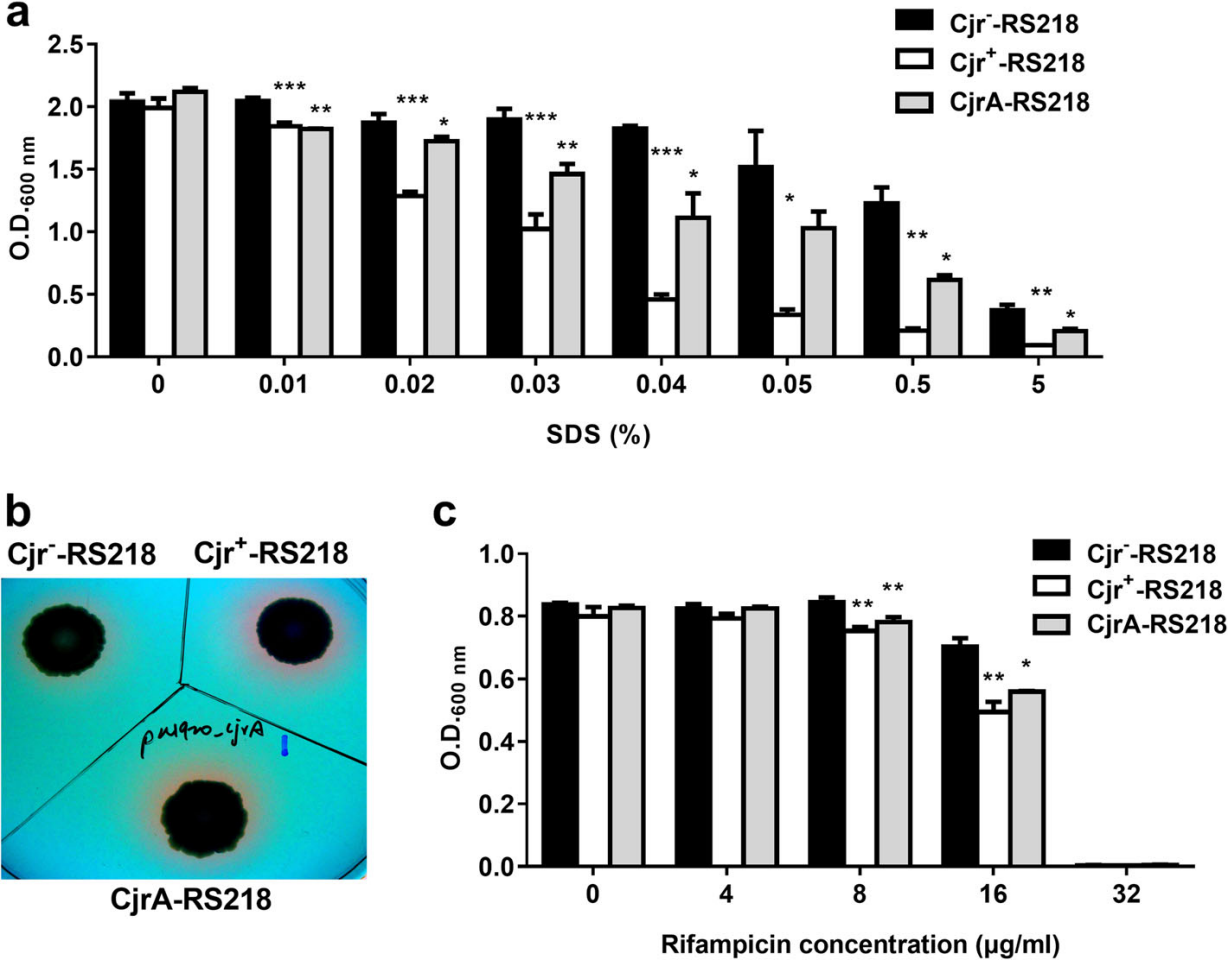
The effects of *cjrABC-senB* expression on the OM properties of the *E. coli* strain RS218. (**a**) SDS sensitivity assays of Cjr^−^-RS218, Cjr^+^-RS218, and Cjr-RS218. The bacterial cultures were adjusted to O.D._600_ = 1 in LB with different concentrations of SDS. After 2 h of incubation at 37 °C, the turbidity (O.D._600_) of each bacterial culture was determined. (**b**) An RNase assay with Cjr^−^-RS218, Cjr^+^-RS218, and CjrA-RS218. The bacteria were grown on an RNase test agar plate. The pink halos around the colonies indicate the leakage of periplasmic RNase into the agar. (**c**) Rifampicin sensitivity assay. Cjr^−^-RS218, Cjr^+^-RS218, and CjrA-RS218 (5 × 10^5^ CFU/ml) were cultured in Mueller-Hinton broth containing different concentrations of rifampicin in a 96 well plate (200 μl/ well). After 8 h of incubation at 37 °C, the turbidity (O.D._600_) of each bacterial culture was determined. For (**a**) and (**c**) the results are shown as the mean ± standard deviation, and the data are representative of three independent experiments performed in triplicate. The asterisks indicate significant differences (*, *P* value < 0.05; **, *P* value < 0.01; ***, *P* value < 0.001) between the O.D._600_ value of the indicated strain and that of Cjr^−^-RS218

The OM of *E. coli* is what the complement system interacts with. It has been shown that OM integrity is important for *E. coli* modulation of complement system activation [18, 23, 38, 39] and for *E. coli* resistance to complement-mediated attack [40]. Because an intact OM is required for bacteria to resist detergent [41, 42], the OM integrity of Cjr^−^-RS218, Cjr^+^-RS218, and CjrA-RS218 was measured through evaluating their detergent resistance. Equal amounts of the bacteria were inoculated into LB with or without different concentrations of SDS (0.01–5%). After 2 h of incubation, we found that Cjr^+^-RS218 and CjrA-RS218 showed significantly lower turbidity (OD_600_) than Cjr^−^-RS218 in the cultures with SDS, while they showed similar turbidity in LB (Fig. 8a). These results indicated that the detergent resistance of Cjr^+^-RS218 and CjrA-RS218 is significantly lower than that of Cjr^−^-RS218, thus suggesting that expressing CjrA or CjrABC-senB may interfere with OM integrity.

In addition, we assessed the abilities of these strains to retain periplasmic proteins, which reflects their OM integrity. A periplasmic RNase assay was performed [31–33]. When grown on RNase test agar plates (see Materials and Methods), RNase leakage from the periplasm formed pink halos around bacterial colonies. As shown in Fig. 8b, Cjr^+^-RS218 and CjrA-RS218 showed more significant leakage of periplasmic RNase than Cjr^−^-RS218, consistently demonstrating that expression of *cjrABC-senB* and *cjrA* compromises OM integrity.

The higher levels of periplasmic protein leakage of Cjr^+^-RS218 and CjrA-RS218 (Fig. 8b) suggest that expression of *cjrABC-senB* and *cjrA* increases OM permeability. OM permeability may affects the sensitivity of bacteria to some antibiotics (eg. Rifampicin). We measured the survival of Cjr^−^-RS218, Cjr^+^-RS218, and CjrA-RS218 in different concentration of rifampicin. As shown in Fig. 8c, the survival of Cjr^+^-RS218 and CjrA-RS218 was significantly lower than Cjr^−^-RS218 in the media containing 8 μg/ml and 16 μg/ml of rifampicin, suggesting that expression of *cjrABC-senB* and *cjrA* may increase bacterial sensitivity to antibiotics.

Finally, we investigated whether *cjrABC-senB* expression affects the expression of the OM components LPS, OmpA, and NlpI and of the periplasmic protease Prc, which have been shown to contribute to ExPEC resistance to the host complement system [18, 23, 43, 44]. However, the expression levels of these factors were not significantly different in Cjr^+^-RS218 and Cjr^−^-RS218 (Additional file 2: Fig. S2a and S2b), suggesting that these bacterial factors are not involved in the decreased serum survival of the *cjrABC-senB*-expressing ExPEC strains.

### The distribution of *cjrABC-senB* in non-UTI-associated *E. coli* bacteremia isolates suggests that the gene cluster does not contribute to human non-UTI-associated bacteremia

In addition to demonstrating that *cjrABC-senB* is epidemiologically associated with *E. coli* UTIs in humans, as mentioned in the “Introduction” section, our previous study, in contrast, shows that the distribution of *cjrABC-senB* is not associated with biliary tract infection (BTI)-associated bacteremia, suggesting that *cjrABC-senB* does not contribute to BTI-associated ExPEC bacteremia in humans [5]. To further investigate whether *cjrABC-senB* is involved in non-UTI-associated and non-BTI-associated bacteremia in humans, 48 *E. coli* bacteremia isolates that are not associated with UTI and BTI were collected and designated non-UBTI bacteremia isolates. The distribution rate of *cjrABC-senB* in the non-UBTI group was determined by PCR. Then, the resulting distribution rate was compared with that in fecal and UTI-associated (cystitis-, pyelonephritis-, and urosepsis-associated) isolates. The distribution rates in the fecal and UTI-associated groups were quoted from the previous study [5]. As shown in Table 2, the frequency of *cjrABC-senB* in the non-UBTI-associated group of isolates was 17%. The frequency in these isolates was significantly lower than those in the UTI-associated isolates, while the distribution showed no significant difference between this group and the fecal group. In combination with the previous finding that *cjrABC-senB* is not associated with BTI-associated bacteremia, these findings suggest that *cjrABC-senB* does not contribute to non-UTI-associated bacteremia (bacteremia not associated with UTIs) in humans. These epidemiological findings are in agreement with the results derived from the mouse model of bacteremia and human serum survival assays in the present study.

**Table 2:** Distributio of *cjrABC-senB* in different source groups of *E. coli* isolates

Gene	No. (%) of *E. coli* isolates					*P*^a^			
	Non-UBTI bacteremia^b^ (*n* = 47)	Fecal isolates (*n* = 115)	Cystitis isolates (*n* = 67)	Pyelonephritis isolates (*n* = 72)	Urosepsis isolates (*n* = 64)	Non-UBTI vs Fecal	Non-UBTI vs Cystitis	Non-UBTI vs Pyelonephritis	Non-UBTI vs Urosepsis
*cjrABC-senB*	8 (17)	23 (20)	24 (36)	32 (44)	23 (36)	–	0.035	0.003	0.033

^a^ Only *P* values < 0.05 (by Fisher’s exact test) are shown

^b^ “Non-UBTI bacteremia” indicates bacteremia isolates that are not associated with UTIs or BTIs

## Discussion

In this study, we demonstrated that *cjrABC-senB*, which has been previously shown to contribute to the pathogenesis of *E. coli* UTIs, hinders ExPEC bloodstream survival. Expression of this gene cluster decreased the pathogen’s ability to resist serum-mediated killing. In NHS, bacteria with *cjrABC-senB* encountered a stronger complement-mediated attack than those without the gene cluster because the harboring strains triggered stronger activation of the complement system through the AP and antibody-dependent CP. Additionally, expression of *cjrABC-senB* increased the pathogen’s susceptibility to complement-mediated attack. Thus, the complement system was responsible for the decreased ability of the *cjrABC-senB*-expressing ExPEC to survive in the bloodstream. Consistently, the molecular epidemiological investigation showed that the distribution of *cjrABC-senB* was not associated with *E. coli*-caused human bacteremia, although it has been previously shown to be associated with *E. coli*-caused human UTIs.

*cjrABC-senB* plays niche-dependent and contradictory roles in the pathogenesis of ExPEC because it contributes to ExPEC UTIs [4] but hinders the bacteria from causing bacteremia. Bacterial virulence factors with similar properties have been reported in other pathogenic bacteria. For example, the PilU protein of *Neisseria meningitidis* contributes to the microcolony formation of the pathogen on host epithelial cells, which is the essential pathogenic step to initiate infection, while expression of this protein decreases the pathogen’s ability to survive in NHS [45]. Additionally, the capsule of *Klebsiella pneumoniae* is known to contribute to bacterial resistance to serum killing and phagocytosis, whereas the capsule impedes pathogen binding to and invasion of epithelial cells [46]. These findings suggest that bacterial virulence factors with niche-dependent and contradictory roles are broadly present in various bacterial pathogens. Identification and understanding of such bacterial factors would help in the development of more precise and efficient therapeutic and preventive measures when utilizing virulence factors as anti-infection targets.

Several studies have suggested that the complement system facilitates ExPEC to cause UTIs, although some other studies have shown that the complement system is functional in urinary tracts (UTs) and thus supposed to facilitate the clearance of invading pathogens in UTs [47–51]. It is reported that mice deficient of the C3 component are resistant to *E. coli* colonization of upper UTs [52]. In addition, opsonization of ExPEC by C3 promotes bacterial binding and invasion of uroepithelial cells [48]. Invasion of uroepithelial cells lining the UTs enhances the survival of infecting *E. coli* by providing protection from host immunity and enable the bacteria to invade into deeper tissues [48]. Given that c*jrABC-senB* facilitates ExPEC colonization of UTs [4] and the distribution of the gene cluster is associated with UTIs (Table 2) [5], the c*jrABC-senB*-induced complement deposition may facilitate *E. coli* to cause UTIs. Li et al. have shown that although the maximum C3 concentration in the urine collected from patients with UTIs is about 1.4% of the C3 concentration in serum. The urine C3 concentration is shown to be sufficient to opsonize *E. coli* in UTs [48]. However, the low C3 concentration in urine suggests that the complement-mediated bactericidal activity in UTs would be significantly lower than that in the serum. It is likely that in UTs *cjrABC-senB* expressing *E. coli* is benefited from the C3 oposonization for binding and invading uroepithelium in UTI, while the effect of the *cjrABC-senB*-raised complement-mediated killing on the bacteria is neglectable.

Our results suggest that Cjr^+^-RS218 can recruit higher levels IgG and properdin deposition, and thus trigger higher levels of CP and AP activation, in comparison with Cjr^−^-RS218. Given that the OM is where the complement system initiate its activation, the compromised OM integrity and increased OM permeability caused by *cjrABC-senB* expression may increase the accessibility of bacterial targets of serum IgG and properdin, enabling higher levels of IgG and properdin binding on bacterial surface and thus triggering higher levels of antibody-dependent CP activation and AP activation. Consistently, a similar mechanistic pattern was found in one of our previous studies showing that a Prc protease mutation of *E. coli* induces compromised OM integrity and increased OM permeability [18]. The Prc mutant can also trigger a higher level of antibody-dependent CP activation in NHS.

In addition, the *cjrABC-senB*-induced interference in the OM integrity may be responsible for the lower survival of Cjr^+^-RS218 in comparison with that of Cjr^−^-RS218 under a similar level of MAC-mediated attack (Fig. 5c). The OM of *E. coli* is where the complement exerts bactericidal activity. It is known that MAC-mediated bactericidal effect occurs through disrupting the OM of *E. coli*, thereby increasing the OM permeability and consequently inducing lethal changes in the inner membrane [53, 54]. The increased OM permeability in Cjr^+^-RS218 may facilitate MAC-mediated bactericidal activity, thus increasing the vulnerability (susceptibility) of the *cjrABC-senB*-expressing bacteria to the MAC-mediated attack.

CjrA was located in the IM fraction of *E. coli*, but its expression affected OM integrity (Fig. 7c). Some IM proteins are known to be involved in maintaining the OM integrity of *E. coli*. For example, the IM proteins TolA, TolQ, and TolR interact with the Pal and TolB proteins to form the Tol-Pal protein complex, which spans the periplasm to link the inner and outer membranes [55]. The intact Tol-Pal complex is required to stabilize the OM. Thus, deletion of these IM proteins compromises OM integrity [56]. It is likely that CjrA may affect OM integrity through interfering with the function of other IM proteins that contribute to OM integrity.

Although among the *cjrABC-senB* genes, *cjrA* is the main factor causing the compromised OM integrity of ExPEC, it may not be fully responsible for the impaired integrity. CjrA-RS218 showed a higher level of SDS resistance than Cjr^+^-RS218 (Fig. 8a), suggesting that, in addition to *cjrA*, other genes in the *cjrABC-senB* gene cluster also contribute to the compromised OM integrity. The subcellular localization of CjrB and CjrC suggests their potential contribution to the compromised OM integrity. CjrB shows significant similarities to TonB and thus is predicted to be a periplasm-exposed IM protein [28]. It is likely that the presence of CjrB in the IM affects the OM integrity in a way similar to that of CjrA. As CjrC is predicted to be an OM protein [28], the presence of CjrA in the OM may directly interfere with the OM structure and thus impair the membrane integrity. *senB* is proposed to play a role in enterotoxin production of enteroinvasive *E. coli* [28]. Its involvement in interfering with the OM integrity cannot be excluded. Additionally, *E. coli* expressing the whole gene cluster (Cjr^+^-RS218) showed significantly lower serum survival than *E. coli* expressing only CjrA (CjrA-RS218). However, expressing CjrB, CjrC, and SenB, respectively, did not decrease bacterial survival in the serum (Fig. 7a). These results suggest that the combined effect of expressing all or multiple of the *cjrABC-senB* genes may be required to induce a level of interference with the OM integrity high enough to further decrease the ability of serum survival, in addition to the effect caused by CjrA alone.

Several lines of evidence suggest that the *cjrABC-senB* gene cluster encodes an iron-uptake system. In addition to the fact that the encoding proteins of the gene cluster are homologous to iron uptake-related proteins (see Introduction), a functional fur box is located upstream of *cjrABC-senB*, and the expression of *cjrABC-senB* is negatively regulated by iron [28], which is a typical iron-dependent regulation mode of iron uptake systems in bacteria [57]. In addition, CjrB and CjrC are known to be involved in colicin Js uptake and are consequently essential for colicin Js sensitivity [28]. It has been known that iron uptake systems are employed to import colicins into sensitive bacterial strains [58]. These findings all indicate that *cjrABC-senB* encodes a potential iron uptake system. However, it remains to be further confirmed whether *cjrABC-senB* facilitates iron uptake.

## Conclusions

Bacterial factors, such as *cjrABC-senB*, with contradictory niche-dependent pathogenic roles may contribute to the development of novel antimicrobial strategies with which we may directly interfere with the proper expression of virulence factors to achieve an antimicrobial effect. For example, increasing *cjrABC-senB* expression in ExPEC may facilitate host elimination of invading ExPEC in the bloodstream. In addition, knowledge of the niche specificity of virulence factors is critical for developing efficient antimicrobial strategies using virulence factors as antimicrobial targets because this knowledge provides information concerning when and where the antivirulence strategy would be the most effective. However, given that most ExPEC virulence genes exist in only a portion of the ExPEC strains and that none of the virulence factors alone is sufficient to account for the virulence properties of the pathogens, an effective and widely usable strategy against ExPEC infections may require a combination of multiple virulence factors as targets. Thus, it is necessary to identify additional virulence factors and understand where and how they contribute to infections.

## Supplementary information

**Additional file 1 Fig. S1.** The fold difference in the serum survival of Cjr^−^-RS218 compared to that of Cjr^+^-RS218 in 40% NHS and 40% NHS with anti-C1q antibody or Anti-properdin antibody treatment. (**a**) The fold difference in the serum survival (the survival rate of Cjr^−^-RS218/the survival rate of Cjr^+^-RS218) after 3 h of incubation in 40% sera, which were NHS, anti-C1q antibody treated NHS, anti-properdin antibody-treated NHS and mannose-treated NHS. The horizontal dashed line represents 1-fold (the survival of the two strains is similar). The serum survival of Cjr^−^-RS218 was approximately 24.8-, 2.2-, 4.1-, and 22.9-fold greater than that of Cjr^+^-RS218 in NHS, anti-C1q antibody treated NHS, anti-properdin antibody-treated NHS and mannose-treated NHS, respectively. (**b**) The fold difference in survival in 40% HI-NHS and 40% of the heat inactivated modified NHS. Cjr^−^-RS218 and Cjr^+^-RS218 showed similar serum survival in these sera. The results are shown as the mean ± standard deviation, and the data are derived from three independent experiments.

**Additional file 2 Fig. S2.** The expression of OmpA, NlpI, Prc, and LPS in Cjr^+^-RS218 and Cjr^−^-RS218 (**a**) The levels of OmpA, NlpI, and Prc in Cjr^+^-RS218 and Cjr^−^-RS218. Equal amounts of bacterial lysates were subjected to SDS-PAGE and then probed with OmpA, NlpI, and Prc antisera. (**b**) LPS of Cjr^+^-RS218 and Cjr^−^-RS218. LPS samples derived from equal amounts of bacteria were analyzed by silver staining after separation by SDS-PAGE.

## Data Availability

All data and materials are fully available and are shown within the manuscript.

## Abbreviations

ExPEC: Extraintestinal pathogenic *E. coli*
UTIs: Urinary tract infections
MACs: Membrane attack complexs
CP: Classical pathway
AP: Alternative pathway
LP: Lectin pathway
CSF: Cerebrospinal fluid
PCR: Polymerase chain reaction
BTIs: Biliary tract infections
NHS: Normal human serum
HI-NHS: Heat-inactivated NHS
CFU: Colony-forming unit
LB: Luria Bertani
IACUC: Institutional Animal Care and Use Committee
DIP: 2, 2′-dipyridyl
PBS: Phosphate buffered saline
LPS: Lipopolysaccharides
hr.: Hour
IM: Inner membrane
OM: Outer membrane
CI: Competitive index
GMFI: Geometric mean fluorescence intensity
CVF: Cobra venom factor
