# Supplementary figures and images for: cjrABC-senB hinders survival of extraintestinal pathogenic E. coli in the bloodstream through triggering complement-mediated killing
Source: J Biomed Sci. 2020 Aug 6;27:86. doi: 10.1186/s12929-020-00677-4 (PMC7412671; doi:10.1186/s12929-020-00677-4)

**a**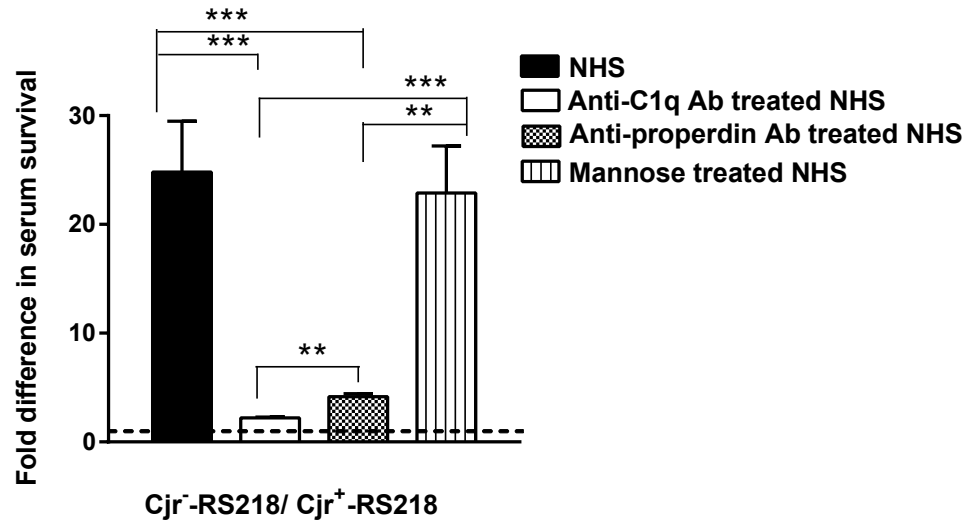**b**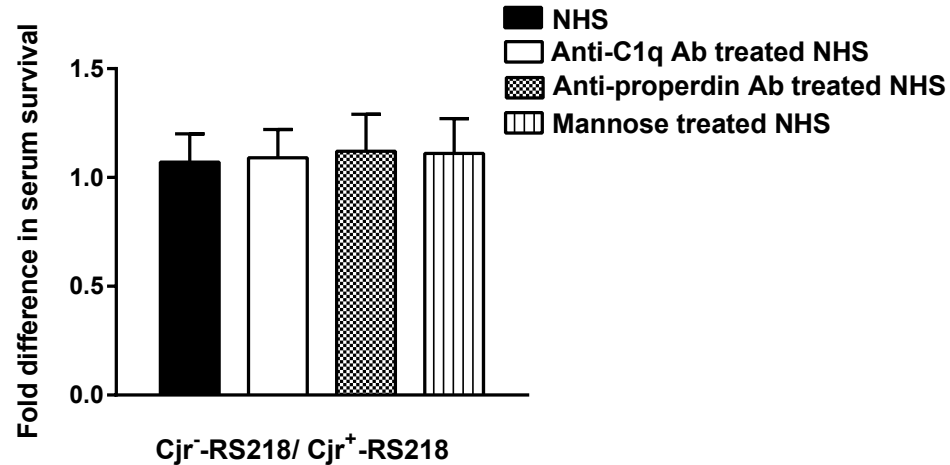

Supplement: Supplementary file 1 — Additional file 1 Fig. S1. The fold difference in the serum survival of Cjr−-RS218 compared to that of Cjr+-RS218 in 40% NHS and 40% NHS with anti-C1q antibody or Anti-properdin antibody treatment. (a) The fold difference in the serum survival (the survival rate of Cjr−-RS218/the survival rate of Cjr+-RS218) after 3 h of incubation in 40% sera, which were NHS, anti-C1q antibody treated NHS, anti-properdin antibody-treated NHS and mannose-treated NHS. The horizontal dashed line represents 1-fold (the survival of the two strains is similar). The serum survival of Cjr−-RS218 was approximately 24.8-, 2.2-, 4.1-, and 22.9-fold greater than that of Cjr+-RS218 in NHS, anti-C1q antibody treated NHS, anti-properdin antibody-treated NHS and mannose-treated NHS, respectively. (b) The fold difference in survival in 40% HI-NHS and 40% of the heat inactivated modified NHS. Cjr−-RS218 and Cjr+-RS218 showed similar serum survival in these sera. The results are shown as the mean ± standard deviation, and the data are derived from three independent experiments. [file 12929_2020_677_MOESM1_ESM.pdf]

**a**

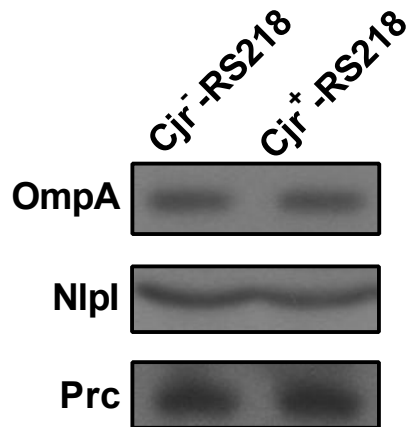

**b**

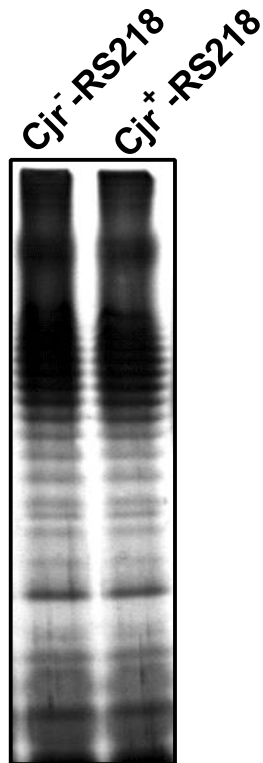

Supplement: Supplementary file 2 — Additional file 2 Fig. S2. The expression of OmpA, NlpI, Prc, and LPS in Cjr+-RS218 and Cjr−-RS218 (a) The levels of OmpA, NlpI, and Prc in Cjr+-RS218 and Cjr−-RS218. Equal amounts of bacterial lysates were subjected to SDS-PAGE and then probed with OmpA, NlpI, and Prc antisera. (b) LPS of Cjr+-RS218 and Cjr−-RS218. LPS samples derived from equal amounts of bacteria were analyzed by silver staining after separation by SDS-PAGE. [file 12929_2020_677_MOESM2_ESM.pdf]
